# Three cases of sperm immobility for intracytoplasmic sperm injection using testicular sperm

**DOI:** 10.1002/iju5.12702

**Published:** 2024-02-09

**Authors:** Noboru Mimura, Teppei Takeshima, Shun Aoki, Tomoki Saito, Jurii Karibe, Yasushi Yumura

**Affiliations:** ^1^ Department of Urology, Reproduction Center Yokohama City University Medical Center Yokohama Kanagawa Japan

**Keywords:** intracytoplasmic sperm injection, Kartagener's syndrome, multiple morphological abnormalities of the sperm flagella, primary ciliary dyskinesia, sperm immobility

## Abstract

**Introduction:**

Sperm immobility is a condition in which sperm are viable but not motile. We reported three patients with sperm immobility, who underwent testicular sperm extraction‐intracytoplasmic sperm injection.

**Case presentation:**

In case 1, a 32‐year‐old patient with sperm immobility had previously undergone intracytoplasmic sperm injection with ejaculated sperm; however, pregnancy was unsuccessful. testicular sperm extraction‐intracytoplasmic sperm injection was performed at our clinic, and pregnancy was achieved. In case 2, a 23‐year‐old patient with clinical varicocele whose semen analysis revealed sperm immobility underwent varicocelectomy, without improvement. Using the hypo‐osmotic swelling test technique, testicular sperm extraction‐intracytoplasmic sperm injection was performed; however, pregnancy was not achieved. In case 3, a 44‐year‐old patient with sperm immobility underwent testicular sperm extraction, and motile sperm were retrieved. testicular sperm extraction‐intracytoplasmic sperm injection using these sperm resulted in pregnancy.

**Conclusion:**

Although testicular sperm extraction‐intracytoplasmic sperm injection is not considered a solution in patients with sperm immobility, pregnancies were achieved. testicular sperm extraction‐intracytoplasmic sperm injection may be successful in some cases in which ejaculated sperm intracytoplasmic sperm injection is unsuitable.

**Keynote message**

In sperm immobility, sperm are not motile. Intracytoplasmic sperm injection using ejaculated sperm with activation is commonly performed; however, the use of testicular sperm may be successful in unsuitable cases.

**Abbreviations & Acronyms**
C‐TESE: conventional testicular sperm extraction
DFI: DNA fragmentation index
EJ‐ICSI: ejaculated sperm intracytoplasmic sperm injection
HDS: high DNA stainability
HOST: hypo‐osmotic swelling test
ICSI: intracytoplasmic sperm injection
IDA: inner dynein arms
IVF: in vitro fertilization
micro‐TESE: microdissection testicular sperm extraction
MMAF: multiple morphological abnormalities of the sperm flagella
ODA: outer dynein arms
PCD: primary ciliary dyskinesia
TESE: testicular sperm extraction

## Background

In sperm immobility, sperm are viable but not motile. Necrozoospermia can be differentiated from sperm immobility. In necrozoospermia, sperm present in the semen are all dead. For diagnosis, one of the methods to differentiate between the two is to stain sperm with eosin Y and check for low sperm viability.

PCD is one of the causes of sperm immobility. The chance of spontaneous pregnancy was very low in patients with PCD.^1^ The first step is to check the viability of immotile sperm obtained by masturbation. Then, ICSI is performed using the ejaculated or surgically retrieved testicular sperm. The sperm are selected using the HOST or adding pentoxifylline to the immotile sperm. Herein, we report three cases in which TESE‐ICSI was performed for sperm immobility at our hospital and review the literature.

## Case presentation

### Case 1

A 32‐year‐old married man visited our department with a complaint of male infertility. His sperm concentration was 1.0 × 10^6^/mL, and the motility was 0% at a previous clinic. Subsequently, he was referred to our hospital after a frozen–thawed embryo transfer using slightly moving sperm failed to result in pregnancy at the previous clinic. He underwent semen analysis at our hospital again, which revealed a semen volume of 2.7 mL, sperm concentration of 17 × 10^6^/mL, and motility of 0.59% with only slight movements. The testicular volume was 20 mL bilaterally without abnormalities. He had no history of chronic bronchitis or bronchiectasis since childhood or findings of a right thoracic heart. C‐TESE was performed, and approximately 1,500 immotile spermatozoa were retrieved. Accordingly, ICSI was performed after a sperm viability test. The HOST method was employed to confirm a sufficient amount of viable sperm, which resulted in the cryopreservation of approximately 2000–3000 sperm cells. Thereafter, the wife underwent oocyte retrieval at the gynecological department. After fertilization, a frozen–thawed embryo transfer of a good cleavage embryo was performed, and the wife achieved pregnancy and live birth.

### Case 2

A 23‐year‐old married man visited our department with a complaint of male infertility. In the previous clinic, he underwent semen analysis twice, and no motile sperm was found in either test. The semen volume was 2.0 mL, his sperm concentration was 22 × 10^6^/mL, one motile sperm was found in all fields of vision, and no sperm with normal morphology was observed. Physical examination revealed a normal testicular volume of 14 mL bilaterally with a grade 2 varicocele on the left side. To improve sperm quality, microsurgical left varicocelectomy was performed. Four months after the surgery, the sperm concentration was 7 × 10^6^/mL, with two motile sperm in all fields of vision; however, no significant improvement was noted in semen findings. Sperm with normal chromatin structure was only 5%, and the rates of fragmented DNA and HDS were 39% and 40%, respectively. Accordingly, TESE‐ICSI was proposed. Genetic examinations revealed no abnormalities. As a result of C‐TESE, numerous immotile sperm with abnormal morphology were retrieved. By using the HOST method, 19 out of 28 viable sperm were found and cryopreserved. ICSI using these sperm was performed; however, all embryos had arrested development and could not be cryopreserved.

### Case 3

A 44‐year‐old married man was referred to our department because his sperm concentration was 1.7 × 10^6^/mL and sperm motility was 0%. Physical examination was normal with a testicular volume of 20 mL bilaterally. After multiple semen examinations, no motile sperm were found. Therefore, ICSI with testicular sperm using pentoxifylline for immotile sperm was scheduled. Only 10 motile sperm were confirmed by C‐TESE and cryopreserved. After frozen–thawed motile sperm were confirmed without pentoxifylline, ICSI and good blastocyst vitrification were performed. A frozen–thawed blastocyst was transferred, resulting in pregnancy and live birth.

## Discussion

In sperm immobility, sperm are viable but not motile. Among others, sperm immobility is caused by PCD, a congenital structural abnormality of airway epithelial cell fibrils and sperm flagella that results in impaired mucus transport, chronic bronchitis, bronchiectasis in children, and male infertility in adults. It is an autosomal recessive genetic disorder with a prevalence of approximately 1 in 20 000.^2^ Kartagener's syndrome, known for its triad of bronchiectasis, chronic sinusitis, and right‐sided heart, is a subset of PCD.^3^ The diagnosis is confirmed by observing the abnormal structure of the sperm flagella and nasal mucosa hairs by electron microscopy in addition to the assessment of history and symptoms of chronic sinusitis and bronchiectasis, which are suspected to be a congenital dysfunction of the cilia.^4^ Particularly, those who do not meet the criteria of PCD but only have sperm flagellar dysfunction are called MMAF. MMAF diagnosis includes electron microscopy of the sperm flagellum ultrastructure to observe axoneme defect, IDA and ODA, and genetic analysis. The known MMAF‐related genes to date include DNAH1, DNAH2 (IDA), and DNAH8 (ODA). There have been reports of pregnancies by ICSI using ejaculated sperm or testicular sperm.^5^

None of these three patients met the classic diagnostic criteria for PCD and were suspected MMAF, but a definitive diagnosis could not be made. In all patients, testicular sperm were confirmed immotile but viable. Previous reports have suggested that ejaculated sperm ICSI (EJ‐ICSI) should be attempted first in cases of sperm immobility, and TESE‐ICSI is not considered a solution because IVF failures in patients with PCD or sperm immobility are attributed to sperm structural abnormalities. Thus, which should be the first choice for such patients is unclear. Therefore, EJ‐ICSI should be tried in the first cycle when sufficient viable sperm can be found, and TESE‐ICSI should be performed in patients with extremely low sperm counts or when EJ‐ICSI does not result in pregnancy.^6^ In our cases, EJ‐ICSI should have been performed after confirming the ejaculated sperm viability; because the spermatozoa were not forward but were very slightly moving, we determined that the patient had sperm immobility and did not perform a viability test. However, even if ICSI is performed in patients with PCD, embryo development is inhibited, resulting in a low probability of pregnancy. The sperm flagellum consists of nine axons and two central microtubules, the same structure as cilia, and the latter is believed to play an important role in fetal development.^7^, ^8^ Genetic analysis was considered for the diagnosis of PCD or MMAF, but was not performed because of the difficulty in its commercial implementation.

We summarized these three cases (Table 1). In case 1, EJ‐ICSI was attempted once; however, pregnancy was not achieved, which led to TESE‐ICSI. Case 2 was preceded by varicocelectomy; however, only immotile sperm with abnormal morphology and chromatin were noted. Therefore, TESE‐ICSI was performed. In case 3, although no motile sperm were observed in the ejaculate, motile sperm were found by C‐TESE, without pentoxifylline. Based on these cases, we believe that TESE‐ICSI may be successful in cases where EJ‐ICSI is unsuitable.

**Table 1:** Summary of three cases of sperm immobility.

Case no.	Patient's age	Wife's age	Semen analysis			Prior treatment	Testicular sperm			Fertilization method	Pregnancy	Live birth
			Semen volume (mL)	Sperm concentration (10^6^/mL)	Motility (%)		Motility	Viability	Viability test			
1	32	36	2.7	1.0	0.0	ICSI	−	+	HOST	ICSI	+	+
2	23	23	2.0	22.0	0.0	−	−	+	HOST	ICSI	−	−
3	44	37	4.2	1.7	0.0	−	+	+	−	ICSI	+	+

## Author contributions

Noboru Mimura: Writing – original draft; writing – review and editing. Teppei Takeshima: Supervision; writing – original draft; writing – review and editing. Shun Aoki: Supervision. Tomoki Saito: Supervision. Jurii Karibe: Supervision. Yasushi Yumura: Supervision.

## Conflict of interest

The authors declare no conflict of interest.

## Approval of the research protocol by an Institutional Reviewer Board

The protocol for this research project has been approved by a suitably constituted Ethics Committee of the institution and it conforms to the provisions of the Declaration of Helsinki. Committee of Yokohama City University Medical Center, Approval No. B210300045. All informed consent was obtained from the subjects.

## Informed consent

The patients provided informed consent for the publication of this case report.

## Registry and the Registration No. of the study/trial

Not applicable.
